# Endogenous hydrogen sulfide mediates the cardioprotection induced by ischemic postconditioning in the early reperfusion phase

**DOI:** 10.3892/etm.2012.733

**Published:** 2012-10-01

**Authors:** YI-E HUANG, ZHI-HAN TANG, WEI XIE, XIN-TIAN SHEN, MI-HUA LIU, XIANG-PING PENG, ZHAN-ZHI ZHAO, DE-BO NIE, LU-SHAN LIU, ZHI-SHENG JIANG

**Affiliations:** 1 Institute of Cardiovascular Disease and Key Laboratory for Arteriosclerology of Hunan Province, University of South China, Hengyang, Hunan 421001;; 2 Huaihua Medical College, Huaihua, Hunan 418000, P.R. China

**Keywords:** hydrogen sulfide, postconditioning, ischemia-reperfusion injury, cardioprotection

## Abstract

Hydrogen sulfide (H_2_S), produced by cystanthionine-γ-lysase (CSE) in the cardiovascular system, has been suggested to be the third gasotransmitter in addition to nitric oxide (NO) and carbon monoxide (CO). The present study aimed to investigate the role of H_2_S in ischemic postconditioning (IPO) during the early period of reperfusion. IPO with 6 episodes of 10 sec reperfusion followed by 6 episodes of 10 sec ischemia (IPO 2’) was administered when reperfusion was initiated. Cardiodynamics and the concentration of H_2_S were measured at 1, 2, 3, 4, 5, 10, 20, 30, 60, 90 and 120 min of reperfusion. Lactate dehydrogenase (LDH) levels and infarct size were determined at the end of the reperfusion. The concentration of H_2_S was stable during the whole experiment in the control group, whereas it reached a peak at the first minute of reperfusion in the ischemia-reperfusion (IR) group. The concentration of H_2_S at the first minute of reperfusion in the IPO 2’ group was higher compared to that of the IR group, which correlated with cardioprotection including improved heart contractile function and reduced infarct size and LDH levels. However, the above effects of IPO 2’ were attenuated by pre-treatment with blockade of endogenous H_2_S production with DL-propargylglycine for 20 min prior to global ischemia. Furthermore, we found that other forms of IPO, IPO commencing at 1 min after reperfusion (delayed IPO) or lasting only for 1 min (IPO 1’), failed to increase the concentration of H_2_S and protect the myocardium. We conclude that the peak of endogenous H_2_S in the early reperfusion phase is the key to cardioprotection induced by IPO.

## Introduction

Ischemic postconditioning (IPO) is defined as a short series of repetitive cycles of brief reperfusion and re-occlusion of the coronary artery applied immediately at the onset of reperfusion. Its cardioprotective effects include the reduction of infarct size and the improvement of coronary artery endothelial dysfunction and neutrophil accumulation in the area at risk (1,2). However, the effects of IPO are determined by the time frame of the early reperfusion. A previous study (3) has reported that the infarct-sparing advantage of IPO and the reduction in malondialdehyde (MDA) and dihydroethidium (DHE) fluorescence intensity were lost when IPO was delayed for the 1-min period of reperfusion. These data suggest that the early moments of reperfusion during which immediate IPO is applied are crucial for its protective effects to take place. Cohen *et al* (4) proposed that the time frame for IPO was the first 2 min at the onset of reperfusion, as 1 min or delayed IPO was ineffective. Hausenloy *et al* (5) suggested that IPO may be mediated through the modulation of the mitochondrial permeability transition pore (mPTP), whose opening in the first few minutes of myocardial reperfusion mediates cell death. The significant burst of reactive oxygen species (ROS) and Ca^2+^ overload during the first minute of reperfusion are important manifestations of ischemic reperfusion injury (6), whereas IPO treatment reduces reperfusion injury by decreasing ROS generation and attenuating mitochondrial Ca^2+^ concentrations. However, the exact mechanisms for IPO warrant further investigation, as the ideal time frame for IPO has not yet been fully elucidated.

Hydrogen sulfide (H_2_S), as an endogenous material, has been characterized as the third gasotransmitter besides nitric oxide (NO) and carbon monoxide (CO) (7). In the cardiovascular system, H_2_S is predominantly generated by cystathionine-γ-lyase (CSE) (7,8). CSE mRNA expression has been reported to be higher in the rat myocardium than in the thoracic aorta, with enzyme activity in the naïve myocardium being 19 nmol/min/g protein (9). The level of H_2_S detected in rat serum was 46 *μ*M (10). Thus, the heart is constantly bathed in a considerable amount of H_2_S generated by the cardiac myocytes. Previously, it has been reported that the concentration of endogenous H_2_S in plasma and myocardial tissue was significantly decreased in isoproterenol-induced myocardial injury (11,12). Additionally, H_2_S has been shown to protect the heart from myocardial ischemia-reperfusion (IR) injury in various studies (13–16). Furthermore, endogenous H_2_S has been shown to mediate the cardioprotection induced by IPO (17). Despite abundant support for H_2_S in the cardioprotective effects of IPO, the involvement of H_2_S in the early reperfusion phase of IPO has not been studied. In the present study, we hypothesized that increased endogenous H_2_S content in the coronary effluent during early reperfusion is indispensible for the beneficial effects of IPO.

## Materials and methods

### Materials

Our study conformed to the Guide for the Care and Use of Laboratory Animals published by the US National Institutes of Health (NIH Publications No. 85-23, revised 1996).

Male Sprague-Dawley rats (230–270 g; n=40) were maintained in an air-filtered, temperature- (20–22°C) and light-(12-h light/dark cycle) controlled room, with a relative humidity of 50–52%. Rats were fed with standard commercial pellets and water *ad libitum*. DL-propargylglycine (PAG) was obtained from Sigma-Aldrich Co. Ltd. (St. Louis, MO, USA).

### Langendorff isolated heart model

**Figure 1:**
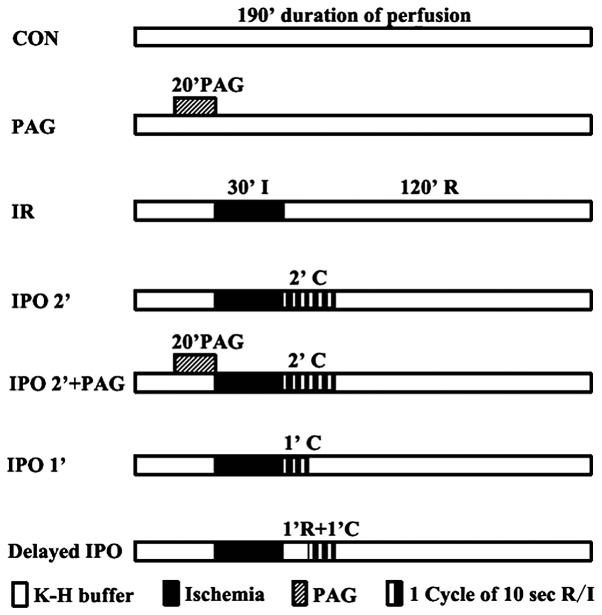
Schematic presentation of the experimental treatment protocol. White field, Krebs solution; black field, no-flow ischemia. DL-propargylglycine (PAG), an inhibitor H_2_S of synthesis, was administered 20 min prior to ischemic treatment. CON, control; IPO, ischemic postconditioning; I, ischemia; R, reperfusion; C, cycles.

Isolated heart experiments were performed as previously described (18). Sprague-Dawley rats were anesthetized with sodium pentobarbital (50 mg/kg). After a midline sternotomy, the hearts were rapidly excised into ice-cold heparinized (5 U/ml) perfusate buffer. After removal of the lung and surrounding tissue, the aorta was rapidly cannulated with a 20-gauge, blunt-ended needle, and retrograde coronary perfusion was initiated at a constant pressure of 80 mmHg with modified Krebs-Henseleit buffer containing: 120 mM NaCl; 25 mM NaHCO_3_; 11 mM D-Glucose; 4.7 mM KCl; 1.2 mM MgSO_4_; 1.2 mM KH_2_PO_4_; and 2.5 mM CaCl_2_, pH 7.4). The perfusate buffer was saturated with a 95% O_2_ and 5% CO_2_ gas mixture at 37°C before use. A latex balloon was inserted into the left ventricle via the left atrium, inflated with distilled water and connected to Maclab System (Maclab, AD Instruments, Ltd., Colarado Springs, CO, USA). The left ventricular end diastolic pressure was set between 5 and 10 mm Hg. The balloon volume was unchanged throughout the experiment. Cardiodynamic function for left ventricular developed pressure (LVDP), rate pressure product (RPP), maximum gradient during systoles (+dP/dt_max_) and minimum gradient during diastoles (−dP/dt_max_) of the heart were continuously monitored with a computer-based data acquisition system. Prior to each experimental protocol, the isolated hearts were allowed to stabilize for 20 min at 37°C. Hearts were excluded from further study if after stabilization they failed to develop steady sinus rhythm or their left ventricular developed pressures were <60 mm Hg. Isolated rat hearts were perfused and stabilized for at least 20 min before recording data. Global ischemia was mimicked by stopping the perfusion of Krebs-Henseleit buffer, i.e., perfusion rate = 0 ml/min. The hearts were randomly divided into 7 groups according to the perfusion protocol as shown in Fig. 1. The control (CON) and PAG groups served as the negative controls in this study. Rat hearts were randomly divided into 7 groups (n=5, each group): i) CON, 190 min duration of perfusion; ii) PAG, treated with 2 mM PAG (the inhibition of endogenous H_2_S synthesis) for 20 min after 20 min stabilization, followed by perfusion for 150 min; iii) IR, after 40 min stabilization, hearts were administered 30 min global ischemia, followed by reperfusion for 120 min; iv) IPO 2’, 6 cycles of 10 sec reperfusion followed by 6 cycles of 10 sec ischemia immediately upon reperfusion (2 min total intervention); v) IPO 2’ + PAG: hearts were pre-treated with 2 mM PAG for 20 min prior to global ischemia and IPO treatment for 2 min; vi) IPO 1’, the algorithm of IPO was repeated for 3 cycles (1 min total intervention); vii) delayed IPO, hearts were reperfused for 1 min, after which the 6 cycles of IPO 2’ algorithm were applied.

### H_2_S concentration measurement

The coronary venous effluent at 1, 2, 3, 4, 5, 10, 20, 30, 60, 90 and 120 min of reperfusion was collected from each group. Samples were diluted with deionised water (final volume, 500 *μ*l) and added to a 1.5-ml tube containing zinc acetate (1% w/v, 250 *μ*l) to trap H_2_S (13,19). Subsequently, N,N-dimethyl-p-phenylenediamine sulphate (20 *μ*M; 133 *μ*l) in 7.2 mol/l hydrogen chloride (HCl) was added, followed by the addition of FeCl_3_ (30 *μ*M; 133 *μ*l) in 1.2 mol/l HCl. Thereafter, trichloroacetic acid (10% w/v, 250 *μ*l) was used to precipitate any proteins present. Samples were cleared by centrifugation (10,000 × g) and the A670 measured on aliquots from the resulting supernatant (300 *μ*l) using an ultraviolet spectrometer 2450 (Shimadzu Corp., Kyoto, Japan). Various concentrations of sodium hydrogen sulfide (NaHS) were used to plot standard curve and calculate the concentration of H_2_S in samples (*μ*mol·l^−1^).

### Infarct size

Infarct size was assessed by triphenyltetrazolium chloride (TTC) staining (20). At the end of the IR protocol, the hearts were cut into 2-mm transverse slices. After incubation in 1% TTC in PBS (pH 7.4) solution for 30 min (37°C), the sections were immersed in formalin (4% w/v) for another 30 min. Images were scanned into a computer and total ventricular area as well as infarct area were determined by computerized planimetry (Adobe Photoshop, version CS3). The infarct size was expressed as a percentage of the total ventricular area (%).

### Lactate dehydrogenase (LDH) content in coronary efflux

The LDH content in the coronary effluent was used as a biochemical marker of cardiomyocyte injury. For each heart, a baseline sample of coronary effluent was collected from the superfusate bath overflow during the final minute of equilibration (prior to the onset of global ischemia). Coronary effluent was then collected at the end of 120 min of reperfusion and a 1.5-ml aliquot was stored on ice for assay within 24 h. LDH content was determined with an automated spectrophotometric clinical assay using an ACE Chemistry Analyzer (Alfa Wassermann Inc., West Caldwell, NJ, USA).

### Statistical analysis

All results are expressed as the means ± SD. Statistical analysis was performed using SPSS 13.0 software for Windows. Differences between groups were analyzed by one-way ANOVA followed by the Student Newman-Keuls test. P<0.05 was considered to indicate a statistically significant difference.

## Results

### Changes in H_2_S concentration in the coronary effluent

**Figure 2:**
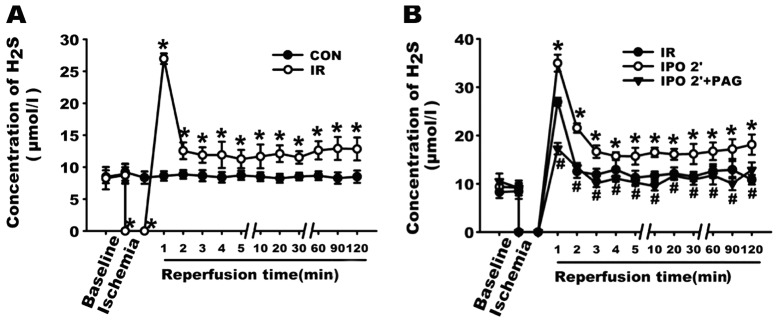
Change in H_2_S concentration (mean ± SD). (A) Effect of ischemia-reperfusion (IR) on H_2_S concentration. (B) Effect of ischemic postconditioning (IPO) 2’ on concentration of hydrogen sulfide (H_2_S) in the presence and absence of DL-propargylglycine (PAG). ^*^P<0.05, compared with control (CON) (A) or IR (B) group; ^#^P<0.05, compared with IPO 2’ group. n=5.

H_2_S is synthesized mainly by CSE in the cardiovascular system. However, it is unclear whether H_2_S can still be generated in ischemic heart. As shown in Fig. 2A, the concentration of H_2_S in the CON group was stable, ranging from 8.28±0.89 to 9.21±1.32 *μ*mol/l. By contrast, after 30-min global ischemia, the concentration of H_2_S in the coronary effluent in the first minute of reperfusion reached a peak (26.96±0.83 *μ*mol/l) of almost 4-fold the level of the baseline, which strongly suggests that during the 30 min of global ischemia, H_2_S was still produced and accumulated in the myocardium. On the basis of this result, we further examined the changes in H_2_S concentration in the coronary effluents. As shown in Fig. 2B, in the first minute after the resumption of flow, the concentration of H_2_S in the IR, IPO 2’, IPO 2’ + PAG group increased to 26.96±0.83, 34.97±1.74 and 17.26±1.20 *μ*mol/l, respectively. Compared with the IR group, the concentration of H_2_S in the coronary effluent was significantly increased in the IPO 2’ group (P<0.05), indicating that the IPO 2’ treatment further increased the level of H_2_S in the coronary effluent during the first minute of reperfusion. However, in the IPO 2’ + PAG group, pre-treatment with PAG for 20 min prior to global ischemia abolished the effect of the IPO 2’ treatment on the content of H_2_S during the first minute of reperfusion. It should be noted that during the entire reperfusion process, the concentration of H_2_S in the coronary effluent in the IPO 2’ group was significantly higher than that in the IR and IPO 2’ + PAG group (all p<0.05).

### Changes in cardiodynamic function

**Figure 3:**
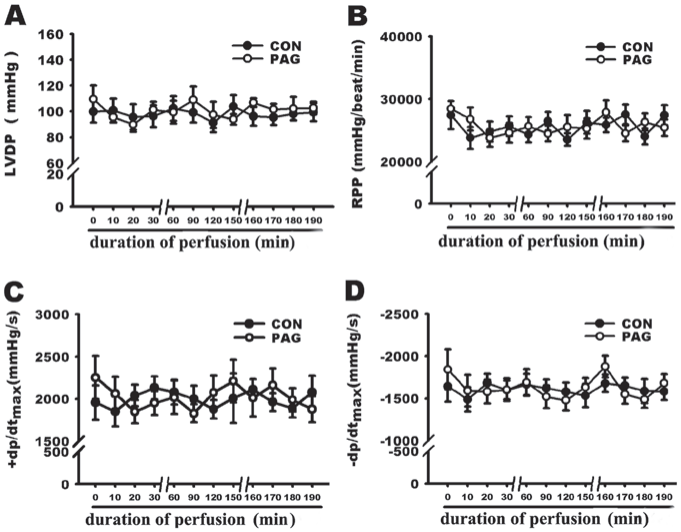
Effect of DL-propargylglycine (PAG) on cardiodynamics (mean ± SD). Mean data of cardiodynamics showing that 2 mM PAG alone did not alter the cardiodynamics significantly. (A) Left ventricular developed pressure (LVDP); (B) rate pressure product (RPP); (C) maximum gradient during systoles (+dP/dt_max_); (D) minimum gradient during diastoles (−dP/dt_max_). n=3 or 4.

The effect of PAG on cardiodynamic function in the isolated rat hearts was examined. As shown in Fig. 3, the administration of PAG (2 mM) alone did not significantly influence cardiodynamic function including LVDP, RPP, +dP/dt_max_ and −dP/dt_max_. PAG was therefore used in the following experiments to determine the involvement of endogenous H_2_S in the cardioprotection induced by IPO 2’ treatment.

**Figure 4:**
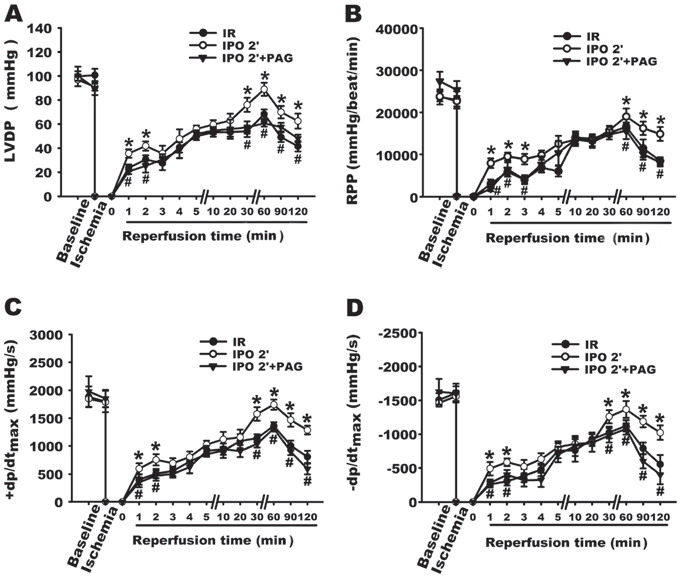
Effect of ischemic postconditioning (IPO) 2’ on cardiodynamics in the presence or absence of DL-propargylglycine (PAG) (mean±SD). (A) Left ventricular developed pressure (LVDP); (B) rate pressure product (RPP); (C) maximum gradient during systoles (+dP/dt_max_); (D) minimum gradient during diastoles (−dP/dt_max_). ^*^P<0.05, compared with ischemia-reperfusion (IR) group; ^#^P<0.05, compared with IPO 2’ group. n=5.

Cardiac mechanical function including LVDP, RPP, ±dp/dt_max_ was measured to determine the involvement of endogenous H_2_S in the cardioprotection induced by the IPO 2’ treatment. As shown in Fig. 4, the IPO 2’ treatment exerted a significant cardioprotective effect with the recovery of cardiac function following ischemia compared with that of IR. The recoveries at 120 min of LVDP, RPP, +dP/dt_max_ and −dP/dt_max_ in IPO 2’ group were 1.50, 1.76, 1.60 and 1.84-fold of the IR group, respectively (all P<0.05 compared with IR, n=5). However, pre-treatment with 2 mM PAG 20 min prior to global ischemia, which inhibited the production of endogenous H_2_S prior to and during ischemia, significantly diminished the cardioprotective effect of the IPO 2’ treatment by reducing LVDP, RPP, +dP/dt_max_ and −dP/dt_max_ to 0.77, 0.55, 0.46 and 0.39-fold of the IPO 2’ group, respectively (all P<0.05 compared with IPO 2’, n=5).

### Changes in infarct size

**Figure 5:**
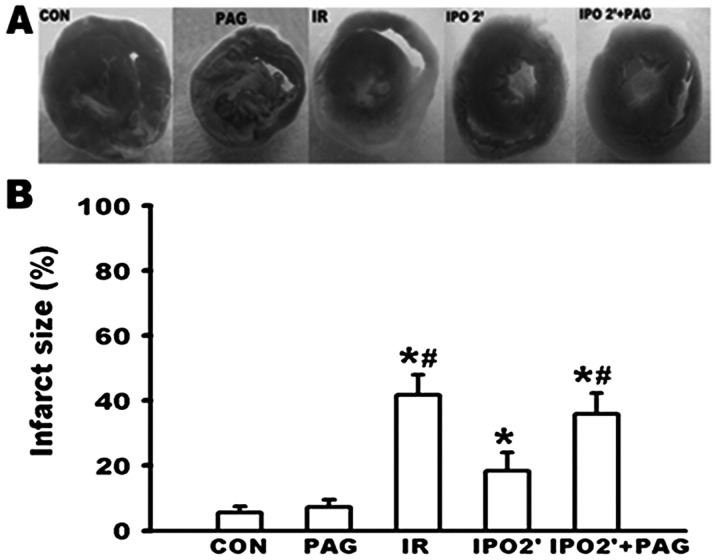
Effect of ischemic postconditioning (IPO) 2’ on myocardial infarction in the presence or absence of DL-propargylglycine (PAG) (mean ± SD). (A) Representative photographs of infarcted heart slices stained with triphenyltetrazolium chloride. Infarcted tissue appears pale, while viable tissue stains red. (B) Mean data of infarct size (expressed as percentage of total ventricular area). ^*^P<0.05, compared with control (CON) group; ^#^P<0.05, compared with IPO 2’ group. n=5 or 6.

As shown in Fig. 5, no significant differences in infarct size were observed between the CON and PAG group. While an infarct size of 41.8±6.2% was caused in the IR group, the infarct size was significantly decreased to 18.3±5.7% in the IPO 2’ group (P<0.05). However, PAG pre-treatment in the IPO 2’ group resulted in an infarct size of 35.9±6.4%. This was significantly increased when compared to the IPO 2’ group (P<0.05), suggesting that PAG inhibited the cardioprotection observed by the IPO 2’ treatment.

### Changes in LDH levels in the coronary effluent

**Figure 6:**
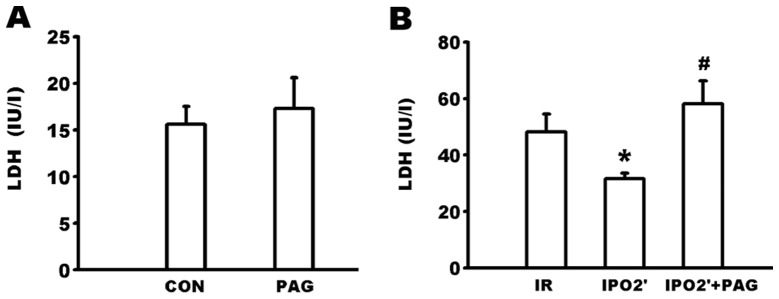
Change in lactate dehydrogenase (LDH) content in the coronary effluent (mean ± SD). (A) DL-propargylglycine (PAG) alone had no significant effect on LDH content. (B) Effect of ischemic postconditioning (IPO) 2’ on LDH content in the presence and absence of PAG. ^*^P<0.05, compared with ischemia-reperfusion (IR) group; ^#^P<0.05, compared with IPO 2’ group. n=5.

As shown in Fig. 6A, there were no significant differences in LDH levels in the coronary efflux between the CON and PAG groups. The LDH content in the coronary effluent at 120 min of reperfusion (Fig. 6B) was significantly lower in the IPO 2’ group compared to the IR group (31.60±1.95 vs. 48.20±6.32 IU/l, P<0.05). However, the LDH level was significantly increased in the IPO 2’ + PAG group (58.20±8.06 IU/l) compared to the IPO 2’ group (P<0.05).

### Effect of IPO 1’ and Delayed IPO

**Figure 7:**
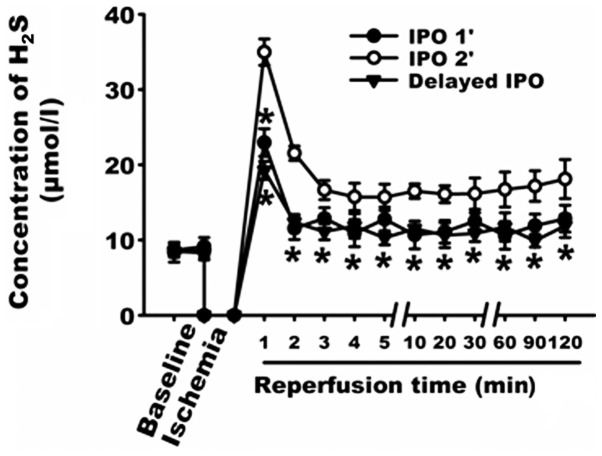
Effect of ischemic postconditioning (IPO) 1’ and delayed IPO on the concentration of hydrogen sulfide (H_2_S) (mean ± SD). ^*^P<0.05, compared with IPO 2’ group. n=5.

**Figure 8:**
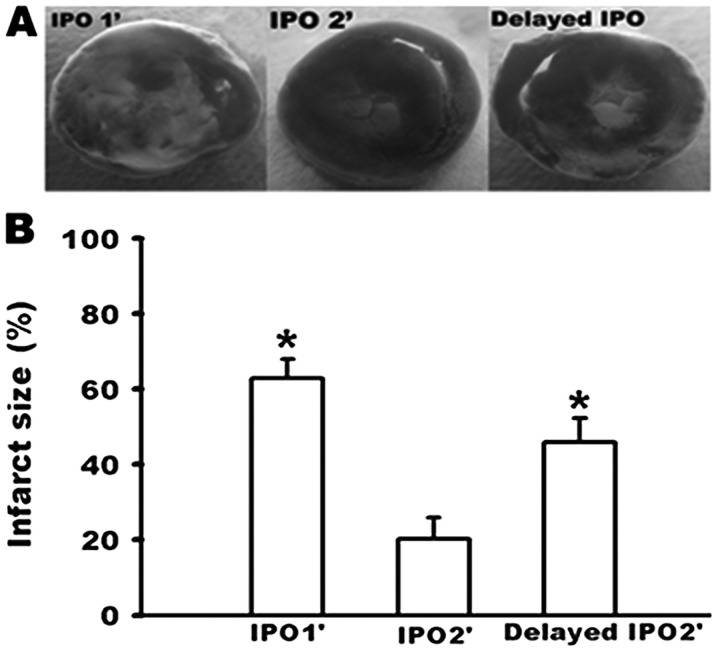
Effect of ischemic postconditioning (IPO) 1’ and delayed IPO on the infart size (mean ± SD). (A) Representative photographs of infarcted heart slices stained with triphenyltetrazolium chloride. (B) Mean data of infarct size (expressed as percentage of total ventricular area). ^*^P<0.05, compared with IPO 2’ group. n=4 or 5.

**Figure 9:**
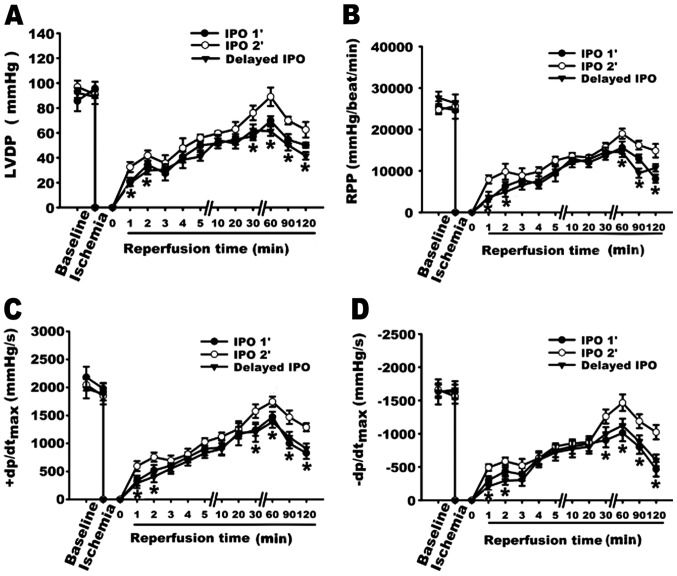
Effect of ischemic postconditioning (IPO) 1’ and delayed IPO on cardiodynamics (mean ± SD). (A) Left ventricular developed pressure (LVDP); (B) rate pressure product (RPP); (C) maximum gradient during systoles (+dP/dt_max_); (D) minimum gradient during diastoles (−dP/dt_max_). ^*^P<0.05, compared with IPO 2’ group. n=5.

As shown in Fig. 7, the concentrations of H_2_S in the IPO 1’ and the delayed IPO groups were decreased when compared to the IPO 2’ group. The cardio-protective effects were reduced in the IPO 1’ and the delayed IPO groups as represented by greater infarct size (Fig. 8) and lower recovery of cardiodynamic function (Fig. 9).

## Discussion

The main objective of this study was to explore the role of endogenous H_2_S in the cardioprotection induced by IPO during early reperfusion. We found that IPO 2’ (IPO lasting for 2 min) significantly improved the heart contractile function and reduced multiple manifestations of IR injury, including infarct size and LDH levels. This is consistent with previous findings that IPO protects the heart from lethal IR injury when assessed by infarct size (3,21,22), cardiodynamic performance (23–26) and cellular injury biomarkers (6,27).

The myocardium generates a considerable amount of H_2_S under physiological conditions (8,9); however, H_2_S production is decreased upon ischemia treatment (19). In the present study, we demonstrated that the concentration of H_2_S was stable throughout the entire experiment in the CON group, and its concentration reached a peak during the first minute of reperfusion in the IR group. This indicates that H_2_S accumulates in the myocardium during the 30 min of global ischemia and is then washed out immediately during early reperfusion. In addition, during the first minute of reperfusion the concentration of H_2_S in the IPO 2’ group was higher than that in the IR group, suggesting that intermittent reperfusion delayed the washout of H_2_S during early reperfusion. On the other hand, H_2_S in aqueous solution is dissociated into HS^−^ and H^+^, which exist in equilibrium with each other. The proportion of undissociated H_2_S under standard temperature conditions at physiological pH (7.4) is 30–33% (20,28), and the proportion increases as pH decreases. As shown in a previous study, acidotic status induced by ischemia is prolonged in IPO 2’ (4), thus the lower pH status induced by IPO 2’ can further increase the concentration of H_2_S at the onset of reperfusion.

It has been previously reported that the early phase of reperfusion may be the key to cardioprotection induced by IPO (3,4). Therefore, we designed other forms of IPO, IPO starting 1 min after reperfusion (delayed IPO) or lasting only 1 min (IPO 1’), to examine the important time frame of IPO 2’. Our data demonstrated that IPO 1’ and delayed IPO failed to exert a cardioprotective effect, and with both treatments the peak of the H_2_S concentration decreased simultaneously during the first minute of reperfusion. There may be 2 explanations for the lower concentration of H_2_S during early reperfusion in both groups. Firstly, there was not enough time for H_2_S accumulation during early reperfusion since IPO 1’ lasted only 1 min. H_2_S was washed out immediately as the reperfusion was initiated, which resulted in reduced retention of H_2_S at the onset of the reperfusion in delayed IPO. Secondly, during early reperfusion in both IPO 1’ and delayed IPO, pH quickly returned to a neutral level. A lower pH is responsible for the generation of increased amounts of H_2_S (4). Indeed, the time-relative changes of H_2_S level are consistent with the critical time frame of the IPO effect. Therefore, our data suggest that IPO lasting only 2 min and commencing at the onset of reperfusion could preserve H_2_S in order to mediate the cardioprotection invoked by IPO.

The accumulation of endogenous material during early reperfusion is one of the important mechanisms of IPO-mediated effects. The observations of Zhao *et al* (2) suggested that endogenous mechanisms are put into action within the first few minutes of reperfusion that attenuate reperfusion injury specifically. Kin *et al* (25) reported that IPO delays the washout of endogenous adenosine during the critical early moments of reperfusion, thereby increasing intravascular adenosine concentrations, which can activate adenosine receptors to elicit protection against myocardial infarction. In addition, previous studies (29,30) have shown that with IPO maneuvers the heart releases autacoids that accumulate in an intermittent manner during early reperfusion and trigger pathways leading to a protected state. In the present study, we noted that the higher concentration of H_2_S during early reperfusion in IPO 2’ was associated with improved recovery of contractile function, decreased infarct size and LDH level. However, the inhibition of endogenous H_2_S synthesis by PAG (the specific and irreversible inhibitor of CSE) used 20 min prior to ischemic treatment notably inhibited the generation of H_2_S and abolished the cardioprotective effects exerted by IPO 2’. Our data indicated that the increase in endogenous H_2_S during early reperfusion contributes to the cardioprotective effects of IPO 2’.

There is a significant burst of oxygen-derived free radicals generated within the first minute of reperfusion peaking 4–7 min following the onset of reperfusion (6), which contribute to the IR injury. H_2_S is a thiol that can interact with and ‘scavenge’ free radicals, including ONOO (31), H_2_O_2_ (12) and HOCl (32). Therefore, the potential cellular mechanisms for H_2_S-mediated early cardioprotection invoked by IPO 2’ may involve the reduction of the peak generation of ROS occurring during the first minutes of reperfusion. In addition, a previous study has shown that H_2_S, the K_ATP_ channel opener, was involved in cardiac protection (33). For this reason we presumed that H_2_S may protect the ischemic myocardum by opening K_ATP_ channels. However, the exact mechanisms remain uncertain in the present study. It is not known whether deleterious mechanisms are attenuated, or whether beneficial mechanisms are triggered by H_2_S. This is a limitation of our present study and warrants further investigation.

In conclusion, this study demonstrates that endogenous H_2_S mediates the cardioprotection induced by IPO during early reperfusion. H_2_S may be considered to protect by ‘pharmacological IPO’ thus offering greater opportunity for protection clinically, such as at the time of thrombolysis, percutaneous transluminal coronary angioplasty (PTCA) and coronary artery bypass grafting (CABG). This study provides an impetus for further investigation into the synthesis of a drug that can release H_2_S. If the certain protection of H_2_S were proved clinically, then ischemia-reperfusion injury could be attenuated by simply administering an oral medication that released H_2_S.
